# N^6^‐methyladenosine (m^6^A) RNA modification in human cancer

**DOI:** 10.1111/cpr.12921

**Published:** 2020-10-07

**Authors:** Fu‐Chun Huo, Zhi‐Man Zhu, Dong‐Sheng Pei

**Affiliations:** ^1^ Department of Pathology Xuzhou Medical University Xuzhou China

## Abstract

N^6^‐methyladenosine (m^6^A) RNA modification, first discovered in 1974, is the most prevalent, abundant and penetrating messenger RNA (mRNA) modification in eukaryotes. This governs the fate of modified transcripts, regulates RNA metabolism and biological processes, and participates in pathogenesis of numerous human diseases, especially in cancer through the reciprocal regulation of m^6^A methyltransferases (“writers”) and demethylases (“erasers”) and the binding proteins decoding m^6^A methylation (“readers”). Accumulating evidence indicates a complicated regulation network of m^6^A modification involving multiple m^6^A‐associated regulatory proteins whose biological functions have been further analysed. This review aimed to summarize the current knowledge on the potential significance and molecular mechanisms of m^6^A RNA modification in the initiation and progression of cancer.

## Graphical abstract

N6‐methyladenosine (m6A) RNA modification in human cancer. m6A modification is a dynamic and reversible process. m6A methylation is catalysed by methyltransferase complexes (writers), reversed by demethylases (erasers) and functionally facilitated by m^6^A‐binding proteins (readers). m^6^A methylation participates in carcinogenesis and tumor progression.

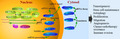

## 1 INTRODUCTION

**Figure 1:**
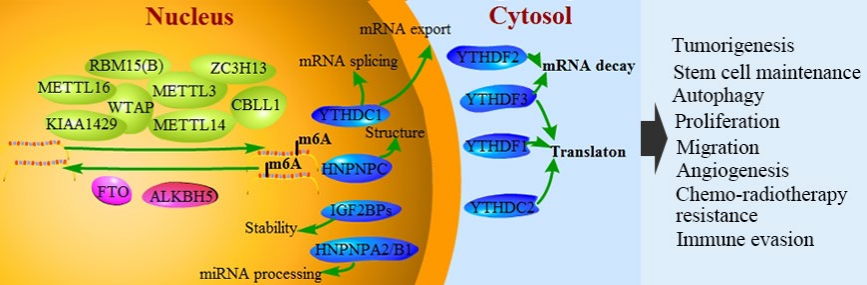
N6‐methyladenosine (m^6^A) RNA modification in human cancer. m^6^A modification is a dynamic and reversible process. m^6^A methylation is catalysed by methyltransferase complex (writers), reversed by demethylases (erasers) and functionally facilitated by m^6^A‐binding proteins (readers). m6A methylation participates in carcinogenesis and tumour progression

More than 170 kinds of chemical modifications have been found in various types of RNAs.^1^ The common examples of RNA modification include N^6^‐methyladenosine (m^6^A), 5‐hydroxymethylcytosine (m^5^C), 1‐methyladenine, N^7^‐methylguanosine, N^6^, 2‐O‐dimethyladenosine (m^6^A_m_), inosine, 8‐oxo‐7,8‐dihydroguanosine, pseudouridine (Ψ), 2′‐O‐methylation and so forth,^2^, ^3^ which confer instinct or complicated manifestations to pathophysiologic changes by embedding additional transcript information into their base sequences. Among these, m^6^A methylation is the primary form of post‐transcriptional modification of RNAs, accounting for 0.1%‐0.4% of adenylation in mammalian RNAs and 50% of all methylated ribonucleotides.^4^, ^5^ m^6^A methylation is installed and exerted effects through methyltransferases (writers), demethylases (erasers) and m^6^A‐binding proteins (readers) to regulate the post‐transcription of genes without altering base sequences.^6^ The profile and characteristic of m^6^A modification remained unidentified due to technical bottleneck and less knowledge about regulators involved, until the first demethylases fat‐mass and obesity‐associated protein (FTO) was reported in 2011, triggering the upsurge of RNA epigenetic transcriptome researches.

m^6^A methylation was discovered in purified poly(A) RNA fraction in 1974.^4^ It is the most pervasive and abundant internal modification in eukaryotic cells, which has been confirmed by other researchers in various eukaryotes, from yeast, Arabidopsis and Drosophila to mammals, and even in viruses. Researchers detected an enormous number of highly conserved m^6^A sites and also determined more than 12 000 m^6^A signal peaks on 7676 mammalian genes by m^6^A sequencing.^8^ m^6^A messenger RNA (mRNA) methylation primarily appears in the consensus motif of RRm6ACH ([G/A/U][G>A]m6AC[U>A>C]) and is enriched in the transcription initiation region, coding sequence (CDS) and 3′‐untranslated regions (3′‐UTR), especially around stop codons of the CDS and the first quarter of 3′‐UTR.^9^ m^6^A methylation is also found near the start codon in *Arabidopsis thaliana*.^10^ In mammals, m^6^A modification exists widely in many tissues, with the highest level of m^6^A modification in the liver, kidney and brain,^9^ showing a character of tissue universality and preference.

m^6^A modification was initially thought to exist only in mRNAs primarily due to the limitations of detection techniques. Subsequent studies found that m^6^A methylation occurred in various types of RNAs, such as small ribosome RNA, transfer RNA, small nuclear RNA (snRNA), small nucleolar RNA, microRNA (miRNA), long non‐coding RNA (lncRNA) and circular RNA.^11^, ^12^ The functions and roles of m^6^A methylation in different biological processes have gained renewed attention. m^6^A modification can regulate RNA metabolism (Figure 1), affect cell fate and functionally participate in a variety of pathophysiological processes, such as stem cell differentiation, cell division, immune homeostasis, mitosis, gametogenesis, sex determination and biological rhythm, and occurrence of numerous human diseases.^13^, ^14^ In this review, starting from m^6^A modification, the related regulators and research progresses on tumours were elaborated, which were expected to become novel markers for molecular diagnosis and potential therapeutic strategies for tumours.

### 1.1 m^6^A‐Writers

The deposition of m^6^A modification is catalysed by a multicomponent methyltransferase complex composed mainly of methyltransferase‐like protein 3 (METTL3), methyltransferase‐like protein 14 (METTL14) and Wilms’ tumour 1‐associated protein (WTAP), which are all the earliest known writer proteins.^15^, ^16^ Subsequently, more new writers, such as methyltransferase‐like protein 16 (METTL16), zinc finger CCCH domain–containing protein 13 (ZC3H13), KIAA1429 (VIRMA), RNA‐binding motif protein 15 (RBM15) and CBLL1 (an E3 ubiquitin ligase) were reported.^1^, ^17^ METTL3 is an S‐adenosylmethionine (SAM)–binding protein and a core component of methyltransferase complex^15^; it can identify potential m^6^A sites and transfer the methyl groups attached on SAM to these sites. METTL14 is another component of the m^6^A methyltransferase complex. Structural studies indicated that METTL3 interacted with METTL14 to form a stable heterodimer structure in the ratio of 1:1 to contribute to recognizing target RNAs.^18^ The physical relationship between METTL3 and METTL14 has a synergistic effect; METTL14 can increase the activity of METTL3 methyltransferase.^18^ Knocking down METTL3 or METTL14 in mouse embryonic stem cells can eliminate up to 99% of total m^6^A level at thousands of sites.^19^ WTAP without catalytic methylation domain and methylase activity is also a component of methyltransferase complex and required for the localization of METTL3‐METTL14 heterodimers to nuclear speckles and the activation of methyltransferase complexes, thereby promoting m^6^A deposition.^18^, ^20^ It is responsible for the recruitment of other m^6^A writers to target RNAs.^18^ ZC3H13 can potentiate the immobilization of WTAP on the nucleus and subsequently strengthen the methyltransferase complexes,^21^ assembling enough complexes for the m^6^A modification. METTL16, recently proposed as a methyltransferase, can bind to U6 snRNA, non‐coding RNA (ncRNA) and precursor messenger RNA (pre‐mRNA), and participate in regulating intracellular homeostasis and mRNA splicing.^22^, ^23^ KIAA1429 is found to catalyse the m^6^A modification. The homologous proteins of KIAA1429 and WTAP in Drosophila melanogaster regulates sex determination by the selective splicing of pre‐mRNAs, affecting Sex‐lethal gene expression.^24^, ^25^ The knockdown of KIAA1429 results in a decrease of in the m^6^A content, which is greater than that achieved by METTL3 and METTL14 knockdown in A549 cells.^26^ RBM15 and its paralog RBM15B can bind to the U‐rich region and catalyse m^6^A modification in some mRNAs and lncRNA XIST.^27^ In 2018, ZC3H13 was identified as a new m^6^A writer in mice and *D melanogaster*.

### 1.2 m^6^A‐Erasers

Until today, only two m^6^A demethylases, FTO and ALKB homolog 5 (ALKBH5), have been identified, both of which belong to the ALKB dioxygenase family and rely on the cofactors Fe^2+^ and α‐ketoglutarate to execute catalytic functions. FTO was initially identified as an obesity‐related gene by genome‐wide association studies.^28^ FTO was the first m^6^A demethylase discovered in 2011, which confirmed that m^6^A modification was a dynamic and reversible process.^7^ FTO affects mRNA stability and translation efficiency by regulating m^6^A modification.^29^ In 2019, FTO was verified to demethylate the m^6^A_m_ modification of snRNA and modulate alternative splicing of mRNAs.^30^ FTO‐mediated m^6^A demethylation involves two steps concomitant with two intermediates, N^6^‐hydroxymethyladenosine and N^6^‐formyladenosine.^31^ ALKBH5 belongs to the ALKB family, but unlike other AIkBs, only ALKBH5 can demethylate m^6^A modification.^32^ ALKBH5 can directly catalyse the methylation of m^6^A‐modified adenosines without producing intermediates.^33^ The expression patterns of FTO and ALKBH5 are different. FTO exists widely in adults and embryos, especially in the brain, while ALKBH5 is expressed in testis. Besides FTO and ALKBH5, more m^6^A demethylases need to be further discovered.

### 1.3 m^6^A‐Readers

m^6^A‐binding proteins mainly include YT521‐B homology (YTH) domain proteins including two subtypes of YTH domain–containing family protein (YTHDFs; YTHDF1/2/3) and YTH domain–containing proteins (YTHDCs; YTHDC1/2); all have a conserved m^6^A‐binding domain and preferentially bind to the consensus RRm6ACH sequence.^27^, ^34^ Besides the YTH domain family, other readers, certain members of the heterogeneous nuclear ribonucleoprotein (HNRNP) family (HNRNPA2B1, HNRNPC and HNRNPG), insulin‐like growth factor 2 mRNA‐binding proteins (IGF2BPs, including IGF2BP1/2/3) and eukaryotic translation initiation factor 3 (eIF3) have been found.^35^, ^36^ Reader proteins displaying a 10‐ to 50‐fold enhancement of m^6^A‐modified mRNA‐binding affinity over unmodified mRNA exercise various downstream effects by interacting with modified RNAs and encode m^6^A modification information.^37^ YTHDF1 promotes the ribosome loading of m^6^A‐modified RNA and improves targeted RNA translation by interacting with translation initiation factors.^34^ YTHDF2 anchors mRNA in decay site processing bodies, inducing mRNA degradation.^38^ Interestingly, YTHDF3 combined with YTHDF1 can enhance mRNA translation, while YTHDF3 combined with YTHDF2 can promote its degradation.^39^, ^40^ However, other studies found that YTHDF2 binding to the m^6^A site prevented FTO from removing m^6^A methylation in the 5′‐UTR region, thereby promoting the cap‐independent translation of mRNAs.^41^ Besides, HNRPNPA2/B1 is involved in the transcription of miRNA precursors (pre‐miRNA).^35^ However, HNRPNPC can affect the secondary structures of mRNAs and lncRNAs.^6^ m^6^A modification can destroy base pairing and improve the accessibility of single‐stranded RNA motifs, thus being recognized by HNRPNPC and HNRPNPG.^6^, ^42^ Although HNRNPC and HNRNPG cannot directly bind to the m^6^A site, they can mediate the selective splicing of m^6^A‐modified transcripts by recognizing and combining m^6^A‐dependent structural switches.^6^, ^43^ Prrc2a has been recently confirmed as an m^6^A reader, and recombinant Prrc2a can stabilize m^6^A‐modified transcripts required for myelin formation.^44^ The newly discovered IGF2BPs are considered to belong to the m^6^A reader family. IGF2BP2 selectively binds to m^6^A‐modified mRNA via K homolog and flanking domains, promoting the translation and stability of mRNA, which is quite different from that of readers with the YTH domain.^45^, ^46^

## 2 M^6^A AND CANCERS

The list of pathophysiology processes regulated by m^6^A modification continues to expand with increasing research and technological breakthroughs. It includes mRNA metabolism, immune modulation, biological rhythm, neural development, autophagy, R‐loop regulation, embryonic and reproductive development and various diseases.^11^, ^47^, ^48^ The m^6^A modification can be traced from systemic lupus erythematosus,^49^ single nucleotide polymorphism (genetic variant),^50^ type 2 diabetes,^51^ inflammatory response and so on.^52^

**Figure 2:**
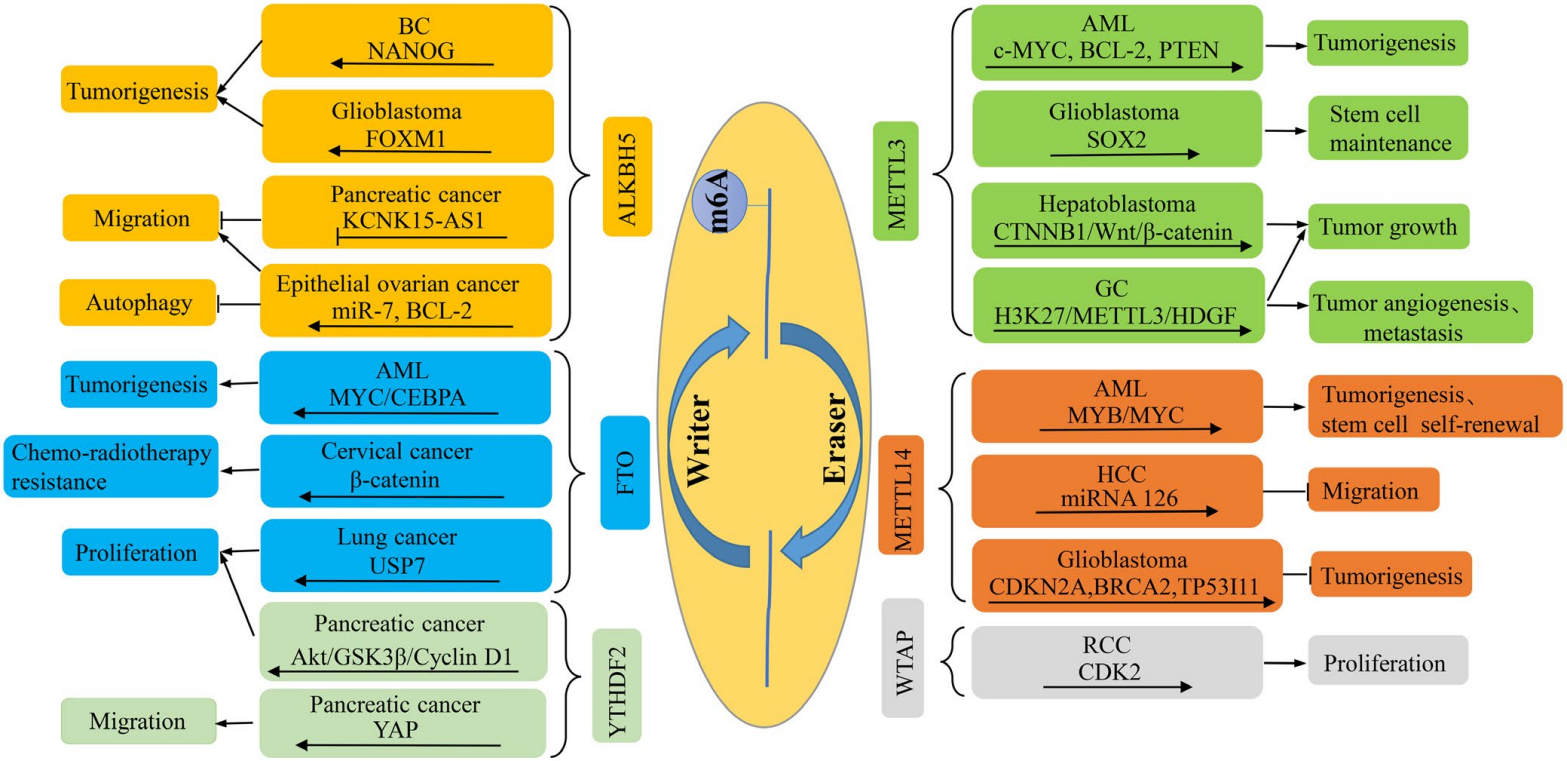
Multiple mechanisms of m^6^A regulators in cancer. Abnormal expression of m^6^A writers, erasers and readers has multiple roles in various types of cancers

Recently, emerging evidence indicates the crucial parts of m^6^A methylation in carcinogenesis and tumour progression by regulating the expression of oncogenes and tumour suppressor genes.^1^, ^14^, ^53^ Analogous to other epigenetic modifications, m^6^A methylation participates in cell proliferation, apoptosis, migration, angiogenesis, autophagy, chemoradiotherapy resistance, energy metabolism, immune escape, self‐renewal and differentiation during the initiation and progression of cancers.^54^, ^55^ Inhibitors of m^6^A regulators have been found and identified as potential inhibitors of cancer progression, suggesting that m^6^A might be a potential target for cancer treatment. The article will review the relationships between m^6^A modification and various types of tumours were discussed further (Table 1, Figure 2).

**Table 1:** Roles of m6A regulators in different cancers

Cancer	m6A regulator	Role in cancer	Mechanism	References
AML	METTL3	Oncogene	Promotes AML progression by promoting the translation of c‐MYC, BCL‐2 and PTEN mRNAs	^57^
	METTL3	Oncogene	Be recruited by CEBPZ to promoters of a specific set of active gene (SP1) and increased translation, resulting in the maintenance of a leukaemic state	^58^
	METTL14	Oncogene	Enhances MYB and MYC expression, promotes myeloid differentiation of HSPCs and AML cells and leukaemogenesis	^56^
	WTAP	Oncogene	Induces abnormal proliferation and arrested differentiation of leukaemia cells	^24^
	FTO	Oncogene	Promotes leukaemic oncogene‐mediated cell transformation and leukemogenesis and weakens the therapeutic effect of ATRA	^62^
	FTO	Oncogene	Be inhibited by R‐2HG, resulting in anti‐tumour effect via targeting FTO/m6A/MYC/CEBPA signalling	^63^
Glioblastoma	METTL3, METTL14	Tumour suppressor	Inhibits GSC self‐renewal and tumorigenesis by down‐regulating oncogenes (ADAM19, EPHA3 and KLF4) and up‐regulating tumour suppressor genes (CDKN2A, BRCA2 and TP53I11)	^68^
	WTAP	Oncogene	Be correlated with glioma grade and poor postoperative survival in glioma patients	^71^
	ALKBH5	Oncogene	Maintains tumorigenicity of GSC by sustaining FOXM1 expression and cell proliferation programme	^72^
BC	METTL3	Oncogene	Promotes cell proliferation via HBXIP/let‐7g/METTL3/HBXIP positive feedback loop	^74^
	KIAA1429	Oncogene	Promotes BC progression by regulating CDK1 by an m6A‐independent manner	^75^
	ALKBH5	Oncogene	Promotes NANOG mRNA and protein expression and mediates enrichment of BCSCs in the hypoxic tumour microenvironment	^76^
	FTO	Oncogene	Promoted cell proliferation, colony formation and metastasis by abrogating the m6A modification of BNIP3 mRNA and epigenetically down‐regulating BNIP3	^78^
HCC	METTL3	Oncogene	Promoted cell proliferation, colony formation and metastasis by abrogating the m6A modification of BNIP3 mRNA and epigenetically down‐regulating BNIP3	^79^
	METTL14	Tumour suppressor	Be inversely correlated with OS and RFS of HCC patients and inhibits tumour metastasis in vitro and in vivo by modulating the pri‐miRNA 126 process in an m6A‐dependent manner via interacting with DGCR8	^81^
	FTO	Oncogene	Be correlated with poor prognosis of HCC individuals and promotes tumorigenesis by mediating demethylation of PKM2 mRNA	^83^
Hepatoblastoma	METTL3	Oncogene	Serves as a diagnostic and prognostic biomarker and promotes tumour growth in vivo by regulating CTNNB1 expression via m6A modification and activating Wnt/β‐catenin signalling	^80^
GC	METTL3	Oncogene	P300‐mediated H3K27 acetylation promotes METTL3 expression, which mediates m6A modification on HDGF mRNA and enhances its stability by an IGF2BP3‐dependent manner, resulting in tumour angiogenesis, GC growth and liver metastasis	^89^
	FTO	Oncogene	Be an increased expression in 375 GC from TCGA data set	^92^
CRC	METTL3	Tumour suppressor	Acts as an independent prognostic factor and suppresses cell proliferation and migration through p38/ERK pathways	^94^
	METTL3	Oncogene	Mediates m6A modification of CBX8 mRNA and promotes its expression, which maintains the stemness and inhibits the chemosensitivity of CRC	^95^
	METTL3	Oncogene	Promotes lncRNA RP11 expression by an m6A‐dependent manner, which targets Siah1/Fbxo45/Zeb1 axis to drive cell dissemination and development of CRC	^96^
	FTO	Oncogene	Low expression of microRNA‐1266 promotes CRC progression via targeting FTO	^97^
	YTHDC2	Oncogene	Be correlated with CRC progression and promotes the cell metastatic ability via promoting translation of HIF‐1α	^99^
Lung cancer	METTL3	Oncogene	miR‐33a or miR‐600 suppresses cell proliferation, migration and invasion by targeting METTL3	101, 102
	ALKBH5	Oncogene	Promotes cell proliferation and invasion by decreasing m6A level on FOXM1 mRNA and increasing its translation under intermittent hypoxia	^106^
	FTO	Oncogene	Promotes cell proliferation by increasing the stability of USP7 mRNA	^104^
	FTO	Oncogene	Promotes progression of lung squamous cell carcinoma by reversing m6A modification of MZF1 mRNA and increasing its stability	^105^
Bladder cancer	METTL3	Oncogene	Be correlated with poor prognosis of bladder cancer patients and promotes cell proliferation in vitro and in vivo and tumorigenesis by modulating the pri‐miR221/222 process in an m6A‐dependent manner via interacting DGCR8	^108^
	METTL3	Oncogene	Promotes bladder cancer progression via AFF4/NF‐kappaB/MYC signalling network	^109^
RCC	METTL14	Tumour suppressor	m6A (METTL14) suppressed P2RX6 activation promotes cell migration and invasion through ATP‐induced Ca2 + influx modulating ERK1/2 phosphorylation and MMP9 signalling pathway	^111^
	WTAP	Oncogene	Be related to poor prognosis of RCC patients and stabilizes CDK2 transcript to enhance CDK2 expression to promote cell proliferation and tumorigenesis in vivo	^112^
Pancreatic cancer	METTL3	Oncogene	Be correlated with poor prognosis of patients with pancreatic cancer and promotes cell proliferation, invasion, migration and chemo‐ and radioresistance	114, 115
	WTAP	Oncogene	Be associated with poor overall survival for pancreatic ductal adenocarcinoma	^116^
	ALKBH5	Tumour suppressor	Inhibits pancreatic cancer motility by decreasing lncRNA KCNK15‐AS1 methylation	^118^
	FTO	Oncogene	Promotes cell proliferation by enhancing stability of c‐Myc mRNA	^117^
	YTHDF2	Unknown	Promotes cell proliferation possibly via Akt/GSK3β/Cyclin D1 pathway and inhibits cell migration and invasion probably via up‐regulation of total YAP	^119^
Cervical cancer	FTO	Oncogene	Contributes to the chemoradiotherapy resistance of cervical squamous cell carcinoma through up‐regulation β‐catenin via mRNA demethylation and the subsequent activation of ERCC1	^122^
	FTO	Oncogene	Be correlated with cervical cancer progression and promotes cell proliferation and migration via controlling m6A modification of E2F1 and Myc transcripts	^123^
Endometrial cancer	METTL3, METTL14	Tumour suppressor	Inhibits cell proliferation, anchorage‐independent growth, and migration and in vivo tumour growth through activation of the AKT pathway	^120^
Ovarian epithelial cancer	ALKBH5	Oncogene	Be related to poor OS and progression‐free survival, promotes cell proliferation and invasion, and inhibits autophagy through the interaction between Bcl‐2 and Beclin‐1 and the EGFR‐PI3K‐AKT‐mTOR pathway	^124^
Cutaneous cancer	METTL3	Oncogene	Promotes colony formation and invasion of melanoma cells by regulating MMP2	^125^
	METTL3	Oncogene	Promotes cSCC cell colony‐forming ability in vitro and tumorigenicity in vivo through modulating ΔNp63 in an m6A‐dependent manner	^126^
Nasopharyngeal carcinoma	METTL3	Oncogene	Mediates m6A modification of ZNF750 mRNA and blocks its expression, which promotes cell growth through regulating FGF14	^127^
Osteosarcoma	METTL3	Oncogene	Promotes cell progression by regulating the m6A level of LEF1 and activating Wnt/β‐catenin signalling pathway	^128^
Cholangiocarcinoma	WTAP	Oncogene	Increases cell motility and tumorigenicity in vivo	^129^

### 2.1 m^6^A in leukemia

METTL3 and METTL14 are highly expressed in acute myeloid leukaemia (AML) cells compared with normal hematopoietic progenitor cells.^56^, ^57^ METTL3 inhibits cell differentiation and apoptosis and promotes cell proliferation in AML.^57^, ^58^ Two possible pathways are responsible for the involvement of METTL3 in the initiation and progression of AML. Firstly, METTL3 increases the expression of the target genes, c‐MYC, BCL‐2 and PTEN, by m^6^A methylation‐mediated translation of mRNAs.^57^ Second, METTL3 combines with the CCAAT‐box‐binding factor of m^6^A‐modified mRNAs in a CEBPZ‐dependent manner, promoting AML deterioration.^58^ METTL14 is essential in the self‐renewal of leukaemic stem/progenitor cells.^59^ In SPI1‐METTL14‐MYB/MYC axis, METTL14 is down‐regulated by SPI1 and enhances MYB and MYC expression through m^6^A modification. It is carcinogenic in enhancing the self‐renewal of haematopoietic stem and progenitor cells (HSPCs) and inhibiting bone marrow differentiation.^56^ RBM15 can maintain the differentiation of HSPCs and megakaryocytic leukaemia cells by controlling mRNA splicing of the crucial differentiation‐related genes including GATA1, RUNX1, TAL1 and C‐MPL.^60^, ^61^ WTAP is also described as a new carcinogen of AML. Increased expression of WTAP promotes cell proliferation and inhibits cell differentiation in AML.^24^ m^6^A writers, METTL3, METTL14 and WTAP, elicit potent oncogenic effects in AML, which provide new thought for AML therapy from a multitude of perspectives.

Most noteworthy, erasers and writers provide complementary mechanisms in AML progression. FTO also acts as an oncogene in AML.^62^ It is highly expressed in some subtypes of AML, such as t (11q23)/MLL‐rearrangement, t (15; 17)/PML‐RARA, FLT3‐ITD and/or NPM1 mutation. It promotes cell proliferation and inhibits differentiation and apoptosis in AML.^62^ It can inhibit all‐*trans*‐retinoic acid (ATRA)–mediated leukaemia cell differentiation and weaken the therapeutic effect of ATRA.^62^ R‐2‐hydroxyglutarate (R‐2HG), the metabolite of isocitrate dehydrogenase 1 and 2 (IDH1/2) mutant, suppresses cell proliferation, induces apoptosis and displays anti‐leukaemic activity by targeting the FTO/m^6^A/MYC/CEBPA axis.^63^, ^64^ Mechanically, R‐2HG binds to FTO and competitively suppresses its demethylase activity, thus resulting in the up‐regulation of global m^6^A modification, which in turn impairs the stability of MYC/CEBPA mRNAs and MYC/CEBPA involving signalling pathways in leukaemia cells sensitive to R‐2HG.^63^, ^64^ FTO depletion reduces the sensitivity of leukaemic cells to R‐2HG. IDH mutations can weaken FTO activity in about 20% of patients with AML; they improve m^6^A modification without affecting FTO expression,^65^ which provides a novel target for the clinical treatment of leukaemia. YTHDF2 is not necessary to maintain the function of hematopoietic stem cells (HSCs); on the contrary, the absence of YTHDF2 helps in enhancing the activity of HSCs.^66^

Currently, conclusions on the m^6^A regulation of tumour progression are inconsistent. METTL3 can bind to the promoter region of target genes and promote translation in an m^6^A‐dependent manner, causing AML deterioration.^58^ However, increased FTO can decrease the tumour suppressor genes ASB2 and RARA and promote the proliferation of AML cells.^62^ Given the inconsistent role of METTL3 and FTO in tumour progression, it is presumed that the discrepancy may be due to METTL3 and FTO acting on different downstream targets. m^6^A modification can regulate the occurrence and development of AML by regulating the mRNAs of critical genes in AML.^67^ Compared with other epigenetic modifications, the m^6^A methylation in AML can be easily targeted chemical medications, thus providing a theoretical basis for the investigation of new drugs against AML.^67^ Inhibiting METTL3/14 may be a novel strategy for the treatment of malignant myeloid tumours.

### 2.2 m^6^A in glioblastoma

At present, the role of METTL3 in the progression of glioblastoma is still controversial. The blockade of METTL3 or METTL14 enhances the proliferation, self‐renewal and tumorigenesis of glioblastoma stem cells (GSCs) concomitant with increased expressions of the ADAM19, EPHA3 and KLF4, and decreased expression of the tumour suppressor genes TP53I11, CDKN2A and BRCA2.^68^ Wang et al reported that m^6^A modification was weaker after knocking out METTL14 in GSCs than knocking out METTL3.^69^ Enforced expression of METTL3 or treatment with FTO inhibitor (MA2) impairs GSC‐initiated tumorigenesis and prolongs the lifespan of GSC‐grafted mice,^68^ implying that METTL3 may serve as an anti‐oncogene in the development of GSCs. Paradoxically, other studies proved that METTL3 was amplified in GSCs and was required for GSC maintenance. METTL3 promotes tumour growth and predicts poor survival in patients with glioblastoma.^70^ Besides, Xi et al^71^ found that increased WTAP is associated with the prognosis in glioblastoma patients. The role of METTL3 and its exact molecular mechanism in GSCs still needs further exploration, which encourages researchers to reveal treatment prospects.

As other m^6^A writers, ALKBH5 participates in the stemness of GSCs. Increased ALKBH5 indicates poor prognosis of patients with glioblastoma while ALKBH5 depletion interferes with self‐renewal ability and inhibits the proliferation and tumorigenesis of GSCs by sustaining FOXM1 expression via ALKBH5‐mediated m^6^A demethylation of FOXM1 nascent transcripts.^72^ In 2019, Chai et al showed that methylation‐related enzymes were closely associated with malignant progression, mesenchymal subtypes and temozolomide sensitivity in glioma through the analysis of RNA sequencing data from 904 glioma samples, providing paramount evidence for the role of m^6^A methylation in glioma progression, prognostic stratification and treatment development.^73^

### 2.3 m^6^A in breast cancer (BC)

Increasing numbers of studies have reported that abnormal m^6^A is involved in BC initiation and progression. HBXIP promotes METTL3 expression through inhibiting tumour suppressor, miRNA let‐7g that targets the 3′‐UTR of METTL3 mRNA. In turn, METTL3 increases the HBXIP level via stimulating its m^6^A modification, which creates a positive feedback loop of HBXIP/let‐7g/METTL3/HBXIP, thus accelerating the malignant proliferation of BC cells.^74^ KIAA1429 regulates CDK1 expression in an m^6^A‐independent manner to promote BC progression.

^75^

Zhang C et al found that HIF‐1α and HIF‐2α determined ALKBH5 expression in BC stem cells (BCSCs) exposed to hypoxia. ALKBH5 enhances NANOG expression by abating m^6^A modification and accelerates the amplification of BCSCs in hypoxic microenvironment,^76^ illustrating the cancer‐promoting function of ALKBH5 in BC. ALKBH5 deletion reverses hypoxia‐induced BCSCs enrichment and tumorigenesis.^72^ Further research showed a complementary role of ALKBH5 and ZNF217 in the negative regulation of m^6^A modification. Although ALKBH5 can induce the m^6^A demethylation of NANOG mRNA, hypoxia‐induced ZNF217 inhibits the m^6^A methylation of NANOG mRNA, maintaining the stability of NONG and KLF4 mRNAs, which ultimately accelerates the malignant progression of BCSCs.^77^ Both of them can elevate the levels of NANOG mRNA and protein, besides enriching BCSCs.^77^ These findings confirmed the role of ALKBH5 in promoting the carcinogenesis of BC. FTO can potentiate the growth and metastasis of BC cells by abrogating the m^6^A modification of BNIP3 mRNA.^78^ It suggests that both writers and erasers of m^6^A seem to show cancer‐promoting effects on BC.

### 2.4 m^6^A in hepatocellular carcinoma (HCC)

Most of the m^6^A‐related enzymes are reported to be up‐regulated in HCC, but METTL14 and YTHDF2 are still debatable. METTL3 is significantly elevated in HCC and associated with poor prognosis in patients with HCC.^79^ METTL3 reduction inhibits cell migration, colony formation and proliferation in vitro and lung metastasis and tumorigenicity of HCC in vivo.^79^ Mechanistically, METTL3 potentiates the degradation of SOCS2 mRNA (tumour suppressor gene) via the m^6^A‐YTHDF2–dependent pathway.^79^ Equally, METTL3‐mediated m^6^A modification activates Wnt/β‐catenin signalling by promoting CTNNB1 expression to drive the growth of hepatoblastoma.^80^ Ma et al demonstrated that an anti‐cancer role of METTL14 in HCC. METTL14 has significantly lower expression in HCC tissues compared with adjacent tissues. Decreased METTL14 expression is associated with poor prognosis and short recurrence time in 130 patients with patients.^81^ Moreover, METTL14 expression has a negative correlation with the proliferation ability of HCC cells.^81^ METTL14 can regulate primary transcripts of miRNA (pri‐miRNA) 126 maturation by interacting with DGCR8. Targeted inhibition of miRNA 126 can reverse the inhibitory effect of METTL14 on HCC progression.^81^ On the contrary, Chen et al found that the expression of METTL14 slightly increased in HCC, and knockdown of METTL14 markedly inhibited the proliferation and metastasis of HCC cells.^79^ KIAA1429 inhibits ID2 expression and promotes the invasion and metastasis of HCC cells.^82^ FTO is linked to an unfavourable prognosis in patients with HCC. It enhances the translation of PKM2 mRNA by relieving m^6^A modification and accelerates HCC aggressiveness.^83^ YTHDF1 is strongly expressed in HCC and closely related to a poor prognosis.^84^, ^85^ Downstream molecules regulated by YTHDF1 are implicated in cell cycle, degradation of amino acids and metabolism of lipids.^84^ m^6^A regulators are more prone to regulate the stability, translation and splicing of mRNA in HCC, and these molecules can be regarded as potential biomarkers and prospective therapeutic targets of HCC.

### 2.5 m^6^A in gastric cancer (GC)

METTL3 can serve as an oncogene‐promoting GC tumorigenesis and progression.^86^, ^87^, ^88^, ^89^ P300‐mediated H3K27 acetylation activation in the METTL3 promoter accounts for the ectopic expression of METTL3 in GC, leading to the enrichment m^6^A modification on HDGF mRNA. IGF2BP3 subsequently recognizes and binds to the methylated HDGF mRNA and augments the stability of HDGF mRNA.^89^ Secreted HDGF drives tumour angiogenesis, while nuclear HDGF elevates ENO2 and GLUT4 levels, followed by a potentiation of the glycolysis ability, resulting in GC growth and liver metastasis.^89^ miRNA‐4429 inhibits GC progression by targeting METTL3 to abrogate the m^6^A‐mediated stabilization of SEC62 mRNA.^86^ Liu et al reported that the expression of mRNAs of METTL3, rather than METTL14, FTO, and ALKBH5, was significantly elevated in GC tissues compared with paired normal tissues; decreased expression of METTL3 suppressed the migration and proliferation of GC cells.^87^ Paradoxically, Xu et al^90^ found that the mRNA and protein levels of FTO were significantly higher in GC tissues than in adjacent tissues. Increased FTO is closely related to poor differentiation, lymph node metastasis, TNM stage and an unfavourable prognosis, and the down‐regulation of FTO inhibits the proliferation, invasion and metastasis of GC cells.^90^, ^91^ The expression of most the 13 widely reported m^6^A regulators, especially FTO, increased in 375 GC cases from the TCGA data set.^91^ Jeopardizing m^6^A modification was associated with oncogene signalling and phenotypes by scrutinizing bioinformatics,^92^ and overriding m^6^A with METTL14 silencing accelerated the proliferation and invasion of GC cells by activating Wnt/PI3K/Akt signalling, which was reversed by FTO depletion.^92^ In GC, low YTHDF2 expression retarded cell proliferation and promote cell apoptosis.^93^ Conclusively, histone modification and ncRNAs affects the expression and regulation of METTL3, contributing to better understand the physiological functions and regulatory mechanisms of METTL3 in cancers. The recent researches mainly concentrate on the role of m^6^A regulators in GC and whether the expression of m^6^A‐related proteins is up‐regulated in the early stage of GC needs further study.

### 2.6 m^6^A in colorectal cancer (CRC)

Increased METTL3 expression acts as an independent and favourable prognostic marker in CRC and inhibits cell proliferation, migration and invasion by activating p38/ERK signalling.^94^ METTL3 potentiates cell stemness properties in vitro and accelerates tumorigenesis and metastasis of CRC in vivo, which can be deciphered by IGF2BP2 that recognizes methylated SOX2 and maintains the stability of SOX2 mRNA.^94^ Similarly, CBX8 potentiates stemness properties and alleviates chemosensitivity to irinotecan (CPT‐11, a camptothecin derivative) and oxaliplatin (L‐OHP, a platinum‐based chemotherapeutic) in colon cancer cells by recruiting Pol II and KMT2b to the LGR5 promoter to enforce its expression.^95^ METTL3‐mediated m^6^A methylation of CBX8 mRNA and IGF2BP1‐induced mRNA stability coordinately sustain CBX expression in colon cancer.^95^ The lncRNA RP11‐targeted Siah1/Fbxo45/Zeb1 axis drives cell dissemination and development of CRC. The up‐regulation of lncRNA RP11 is implemented partly by m^6^A modification.^96^ Shen et al^97^ demonstrated that FTO was highly expressed in CRC tissues and negatively regulated by miRNA 1266. Collectively, it can be certain that METTL3 plays important role in CRC progression, but whether it serves an oncogene or a tumour suppressor gene is controversial.

YTHDF1 is associated with malignant phenotypes in CRC. The blockade of YTHDF1 suppresses cell proliferation and enhances the chemosensitivity of cancer cells to 5‐fluorouracil (5‐FU) and L‐OHP. Oncogene MYC promotes the transcriptional activation of YTHDF1 rather than other YTH domain family members.^98^ Immunohistochemical staining showed that YTHDF2 positively correlated with the stage of CRC.^99^ YTHDF2 promotes the metastasis of CRC by promoting the HIF‐1α translation, and knocking out YTHDF2 can weaken HIF‐1α expression and inhibit the metastasis of CRC cells in vitro and in vivo.^99^ Liu et al recently discovered that most of the m^6^A regulators, except YTHDC2, showed significant differences in comparing of cancer tissues and adjacent mucosal tissues. Higher HNRNPC and lower YTHDF1 expression may act as the prognostic predictors of poor overall survival (OS) in colon cancer through excavating the TCGA data set including 331 colorectal adenocarcinoma samples.^100^

### 2.7 m^6^A in lung cancer

miRNA‐33a suppresses the proliferation of non–small cell lung cancer (NSCLC) cells via targeting 3′‐UTR of METTL3.^101^ METTL3 silencing expedites cell apoptosis via AKT signalling pathway and inhibits the proliferation, migration and invasion of lung cancer cells. miRNA‐600 reverses METTL3‐induced tumorigenesis and progression of lung cancer.^102^ Lin et al^103^ also confirmed that METTL3 promoted cell growth, proliferation and invasion in lung adenocarcinoma. Besides being an m^6^A “writer”, METTL3 may serve as an m^6^A “reader” in the cytoplasm by identifying and interacting with translation initiation factor, enhancing the translation of EGFR and TAZ in lung cancer.^103^ It indicates that METTL3 conveys oncogenic signals to potentiate aggressiveness in lung cancer.

FTO acts as a proto‐oncogene in NSCLC.^104^ The knockdown of FTO inhibits the proliferation of lung cancer cells by modulating the mRNA stability of USP7 via FTO‐mediated m^6^A demethylase.^104^ Furthermore, FTO rather than METTL3, METTL14 and ALKBH5 is considered as the main factor causing the dysfunction of m^6^A modification in lung squamous cell carcinoma.^105^ FTO can enhance the stability of MZF1 mRNA by reversing m^6^A modification, inducing MZF1 expression and ultimately promoting the progression of lung squamous cell carcinoma.^105^ All these indicate that FTO may be a potential therapeutic target in lung cancer. Intermittent hypoxia‐induced ALKBH5 amplification in lung adenocarcinoma promotes cell proliferation and invasion by decreasing the m^6^A level of FOXM1 mRNA and increasing the translation of FOXM1 mRNA.^106^

### 2.8 m^6^A in bladder cancer and renal cell carcinoma (RCC)

Enrichment of m^6^A modification is associated with bladder cancer. Bioinformatics analysis revealed that m^6^A regulators were associated with the malignant progression of bladder cancer.^107^ METTL3 may serve as an oncogene in bladder cancer, contributes to cell proliferation and drives tumorigenesis, which is mediated by interacting with DGCR8 and subsequently accelerating the pri‐miR221/222 process in an m^6^A‐dependent manner concomitant with PTEN down‐regulation, and METTL3 correlates with poor prognosis and may be proposed as a potential therapeutic target for bladder cancer.^108^ The ectopic expression of METTL3 potentiates bladder cancer progression through the AFF4/NF‐κB/MYC network.^109^

m^6^A abnormity plays a crucial role in ccRCC. PI3K/mTOR and p53 pathways may function as downstream targets of m^6^A modification in RCC.^110^ ATP/P2RX6 modulates Ca^2+^ influx–mediated MAPK ERK1/2 phosphorylation and MMP9 signalling to potentiate the migration and invasion of RCC cells.^111^ Noteworthy, METTL14 is down‐regulated and abrogates P2RX6 expression through modulating m^6^A modification, which affects the malignant progression of RCC.^111^ WTAP activates the proliferation of RCC cells by regulating the stability of CDK2 mRNA, leading to the occurrence and development of RCC; patients with RCC and overexpression of WTAP have a dismal prognosis.^112^ According to the latest research, transcriptome‐wide m^6^A patterns in clear cell RCC (ccRCC) tissues are significantly different from that of neighbouring non‐cancerous tissues. A total of 6919 new m^6^A peaks appeared concurrent with the disappearance of 5020 peaks in ccRCC samples were compared with non‐cancerous tissues; the unique distribution profile of m^6^A regulated gene expression and pathways in ccRCC.^113^

### 2.9 m^6^A in pancreatic cancer

The expression of METTL3 increases at both protein and mRNA levels in pancreatic cancer; METTL3 knockdown inhibits cell proliferation, invasion and migration.^114^ Pancreatic cancer cells with METTL3 silencing are more susceptible to irradiation, gemcitabine, 5‐FU and L‐OHP, but cell morphology and proliferation are not affected.^115^ METTL3 and METTL14 are proposed as novel and promising targets for enhancing chemosensitivity and radiosensitivity of pancreatic cancer cells.^115^ In pancreatic cancer, METTL3 correlates with carcinogenesis and may act as a potential therapeutic target.^114^, ^115^ In pancreatic ductal adenocarcinoma, increased WTAP expression is significantly related to the sex and tumour stage and appears to be an independent and valid prognostic factor.^116^ FTO markedly promotes cell proliferation, suppresses cell apoptosis and enhances mRNA stability of c‐MYC in pancreatic cancer.^117^ Low expression of ALKBH5 in pancreatic cancer cells reverses the m^6^A modification of lncRNA KCNK15‐AS1, resulting in a decrease in the ability of cancer cells to invade and metastasize.^118^

The expression of YTHDF2 is up‐regulated in pancreatic cancer and significantly associated with tumour stage.^119^ YTHDF2 has a reverse regulatory effect on cell proliferation and invasion in pancreatic cancer. The down‐regulation of YTHDF2 reduce the cloning density and proliferation curve by regulating Akt/GSK3β/cyclin D1 pathway.^119^ However, the down‐regulation of the expression of YTHDF2 promotes cell migration, invasion and EMT through the YAP signalling pathway.^119^ The relationship between YTHDF2 and YAP in pancreatic cancer cells needs further exploration, especially whether the reverse regulation of YTHDF2 exists in other tumours.

### 2.10 m^6^A in gynecological cancer

m^6^A modification is frequently found at low levels in gynaecological cancers, such as endometrial cancer,^120^ cervical cancer^121^, ^122^, ^123^ and epithelial ovarian cancer.^124^ Low m^6^A level, as an independent prognostic indicator, was found to be associated with cancer progression and poor outcome through screening 286 cervical cancer samples, and augmenting m^6^A modification with the FTO inhibitor MA2 suppressed the tumour development in mouse models.^121^, ^122^ FTO is frequently overexpressed in cervical cancer and associated with cervical cancer progression.^121^, ^123^ Patients with cervical cancer and co‐expression of FTO and β‐catenin have a worse prognosis.^122^ FTO contributes to the proliferation and migration of cervical cancer cells by directly interacting with E2F1 and MYC transcripts and impairing their translation efficiency in a demethylase‐dependent manner.^123^ Overall, it offers the possibility of FTO for a clinical diagnostic and therapeutic target in cervical cancer.

In 2018, Liu et al observed that 70% of endometrial cancer exhibited decreased m^6^A methylation probably through either METTL14 (R298P) mutation or METTL3 down‐regulation.^120^ Reduced m^6^A methylation is accompanied by increased expression of mTORC2 and decreased expression of PHLPP2 and potentiates cell proliferation and invasion in endometrial cancer partly by activating the AKT pathway.^120^ Through abolishing m^6^A methylation, ALKBH5 enhances the stability of Bcl‐2 mRNA, potentiates the interaction between Bcl‐2 and Beclin‐1, and activates the EGFR/PIK3CA/AKT/mTOR pathway to promote the proliferation, invasion and autophagy of ovarian epithelial cancer cells.^124^

### 2.11 m^6^A in cutaneous cancer

METTL3 functions as an oncogene in cutaneous cancer.^125^, ^126^ Its expression increases in cutaneous squamous cell carcinoma (cSCC). Administration of cSCC cells with the methylation inhibitor cycloleucine or silencing METTL3 expression affects the colony‐forming efficiency in vitro and carcinogenesis in vivo partly through modulating ΔNp63 in an m^6^A‐dependent manner.^126^ METTL3 promotes cell invasion and migration and increases MMP2 and N‐cadherin levels in melanoma cells, which are completely reversed in cells transfected with inactivated METTL3 through site mutant.^125^ Inhibiting m^6^A methylation may provide a good prospect in the treatment of melanoma.

### 2.12 m^6^A in other tumors

Zhang et al uncovered that the ZNF750/FGF14 axis accelerated cell apoptosis and inhibited the growth of nasopharyngeal carcinoma in vitro and in vivo; METTL3‐dependent m^6^A modification was enriched in the CDS of ZNF750 mRNAs and impaired ZNF750 expression,^127^ thus proving the oncogene role of METTL3 in nasopharyngeal carcinoma. The m^6^A level in total RNA increased in osteosarcoma tissues and cell lines, and METTL3 promoted the cell proliferation, migration and invasion of osteosarcoma by modulating the m^6^A level of LEF1 and activating Wnt/β‐catenin.^128^ WTAP is highly expressed in cholangiocarcinoma, especially in cholangiocarcinoma cells with metastasis to lymph nodes or vessels. The overexpression of WTAP can significantly increase the metastasis and invasion of cholangiocarcinoma cells.^129^

## 3 M^6^A AND RELATED INHIBITORS

Considering that epigenetics is characterized by regulating transcription and post‐transcriptional products, previous studies showed that m^6^A methylation has crucial role in malignant biological behaviours. Therefore, developing specific inhibitors of m^6^A‐related proteins is of great scientific significance and clinical value, thus being vital in tumour therapy.^130^, ^131^, ^132^, ^133^ Gemcitabine has emerged as an inducer of apoptosis in pancreatic cancer cells with low METTL3 expression.^115^ CRC cells with YTHDF1 silencing are more sensitive to L‐OHP and 5‐FU.^98^ METTL3 drives the ectopic expression of CBX8 in an m^6^A‐dependent manner, which obviates the sensitivity of colon cancer cells to chemotherapy of CPT‐11 and L‐OHP.^95^ Single medication is prone to produce drug resistance in the clinical application. The aforementioned findings on m^6^A methylation and chemotherapeutic drugs provide a promising strategy for tumour therapy.^1^, ^134^

At present, the development of inhibitors based on m^6^A‐related enzymes is focused mainly on the first discovered RNA demethylase FTO. Yang et al proposed that Rhein might serve as a competitive inhibitor of FTO through the inhibition of the catalytic domain of FTO.^131^ Subsequently, fluorescein derivatives,^135^ MA (a non‐steroidal anti‐inflammatory drug),^132^ IOX3^136^ and radicicol ^133^ were also identified to decrease FTO expression. MA, by competently binding to FTO at the m^6^A site, can significantly increase the m^6^A level without affecting the demethylase activity of ALKBH5.^132^ At the same time, MA2, an ethyl esterification isomer of MA, possesses better cell penetration ability and contributes to m^6^A modification, thus providing a chemical basis and guidance for the development of specific FTO inhibitors. Moreover, N‐CDPCB, CHTB and entacapone can also exert potent suppressive activities against FTO.^130^, ^133^, ^137^, ^138^ As the first obesity‐related gene identified by genome‐wide association analysis analysis,^28^ not only FTO is closely associated with obesity and tumour, but also its common variant rs9939609 may be associated with central nervous system diseases, such as brain volume loss and alcohol dependence.^139^ Therefore, FTO inhibitors may also be developed as drugs for neurological diseases, besides their anti‐cancer and weight‐reducing effects in addition to anti‐tumour and weight loss, may also be developed as drugs for neurological diseases. The progress on methyltransferase inhibitors is relatively slow. So far, only 3‐deazaadenosine has been proven to inhibit METTL3, but it has a broad‐spectrum efficacy and inhibits the activity of all m^6^A “readers”.^140^ Furthermore, chemical oxidation eliminates the m^6^A modification of mRNA.^141^, ^142^ Although several m^6^A demethylase agents have been reported in the literature, their specificity or efficacy cannot achieve the goal of precise and suboptimal treatment. Precise and effective m^6^A targeted drugs still need further research and development.

## 4 CONCLUSIONS AND PERSPECTIVES

Various biological processes and diseases have been revealed in detail with increasing investigations on the composition of m^6^A regulatory network and its significance in mRNA processing and metabolism.^143^ As the most prevalent modifications of mRNAs, m^6^A modification, characterized by widespread existence, unique distribution and dynamic reversibility, is involved in almost the whole course of mRNA biology from production to degradation. It participates in the regulation of biological functions of miRNAs and lncRNAs,^12^, ^144^ which acts as an important regulator of stress response, biological rhythm, cell differentiation, immune response, virus replication and infection, adipogenesis, embryonic development, sex determination, carcinogenesis, tumour progression, and so on.^14^, ^54^

Many issues related to the function and action mechanism of m^6^A methylation remain unresolved. Novel m^6^A readers and other components of m^6^A methyltransferase complex have emerged successively. No eraser has been uncovered except the known ALKBH5 and FTO. Considering the reciprocal effects on mRNA stability and degradation, the m^6^A reader‐mediating biological functions warrant further exploration. The rapid development of the detection technology of m^6^A methylation (sites) can make it easier to clarify the m^6^A regulatory network. Although several inhibitors of m^6^A‐related factors have been discovered, clinical applications have a far way to go. Effectuating the dynamic monitoring and tracking of m^6^A methylation is a fundamental issue confronting researchers.

From the epitranscriptome perspective, the tissue‐specific and inhomogeneous distribution of m^6^A modification provides a new direction for comprehending the pathogenesis in numerous diseases, especially in tumours. m^6^A modification, as a “double‐edged sword”, can promote or inhibit the initiation and progression of tumours mainly by regulating mRNA levels of oncogenes or tumour suppressor genes. It regulates cell proliferation, migration, invasion, differentiation and sensitivity to radiochemotherapy. However, whether m^6^A modification is implicated in a tumour microenvironment has not been reported. Further studying the molecular mechanisms of m^6^A methylation and its regulators can shed light on the clinical diagnosis and targeted therapy of tumours.

## CONFLICT OF INTEREST

The authors declare that there is no conflict of interests.

## AUTHOR CONTRIBUTIONS

FCH and ZMZ wrote and discussed the manuscript. DSP designed and drafted the manuscript.

## Data Availability

The data that support the findings of this study are available from the corresponding author upon reasonable request.
